# Economic Evaluation of FebriDx®: A Novel Rapid, Point-of-Care Test for Differentiation of Viral versus Bacterial Acute Respiratory Infection in the United States

**DOI:** 10.36469/001c.27753

**Published:** 2021-09-30

**Authors:** Katherine Dick, John Schneider

**Affiliations:** 1 Avalon Health Economics

**Keywords:** acute respiratory infection, antibiotic resistance, point of care, diagnostic, antibiotic stewardship

## Abstract

**Background:** Acute respiratory infections (ARIs) are commonly treated with antibiotics in outpatient settings, but many infections are caused by viruses and antibiotic treatment is therefore inappropriate. FebriDx®, a rapid point-of-care test that can differentiate viral from bacterial infections, can inform antibiotic treatment decisions.

**Objectives:** The primary aim of this study is to conduct a literature-based US economic evaluation of a novel rapid point-of-care test, FebriDx®, that simultaneously measures two key infection biomarkers, C-reactive protein (CRP) and Myxovirus resistance protein A (MxA), to accurately differentiate viral from bacterial infection.

**Methods:** A budget impact model was developed based on a review of published literature on antibiotic prescribing for ARIs in the United States. The model considers the cost of antibiotic treatment, antibiotic resistant infections, antibiotic-related adverse events, and point-of-care testing. These costs were extrapolated to estimate savings on a national level.

**Results:** The expected national cost to treat ARIs under standard of care was US 8.25billion,whereastheexpectednationalcostofFebriDxpoint−of−care−guidedARItreatmentwasUS5.74 billion. Therefore, the expected national savings associated with FebriDx® rapid point-of-care testing was US $2.51 billion annually.

**Conclusions:** FebriDx, a point of care test that can reliably aid in the differentiation of viral and bacterial infections, can reduce antibiotic misuse and, therefore, antibiotic resistant infections. This results in significant cost savings, driven primarily by the reduction in antibiotic resistant infections.

## INTRODUCTION

Acute respiratory infections (ARIs) are common in outpatient settings and account for 41% of all antibiotics prescribed in ambulatory settings, about half of which are medically unnecessary.^1,2^ Common ARIs treated in outpatient settings include sinusitis, otitis media, bronchitis, and pharyngitis. Many of these infections are caused by viruses but are inappropriately treated with antibiotics.^1,3^ This misuse of antibiotics has serious consequences, including the emergence of antibiotic resistant infections and drug-related adverse events. According to the Centers for Disease Control, 2.8 million antibiotic resistant infections occur annually in the United States, 35 000 of which are fatal.^4^ An additional 12 800 fatalities are caused by antibiotic-associated *Clostridium difficile* infections.^4^ Antibiotic-related adverse events are a common cause of both hospitalizations and emergency department (ED) visits, accounting for over 16% of all outpatient adverse drug event visits.^5^

Antibiotic misuse is a significant economic burden on the health-care system. US antibiotic expenditure totaled US 8.8billionin2015,themajorityofwhichwasattributabletoantibioticprescribinginoutpatientsettings.[@87640]AntibioticresistanceisestimatedtocosttheUSeconomyanadditionalUS20 to US $50 billion annually.^1,3^ Reducing unnecessary antibiotic use could have a significant impact on morbidity, mortality, and overall health-care costs related to antibiotic resistance and adverse drug events.^1,4^ Key drivers of antibiotic misuse include difficulty differentiating viral from bacterial infection due to overlapping clinical presentation and long laboratory-based testing turnaround times.^1,6^ This diagnostic uncertainty, coupled with clinician concern over missing bacterial infections and patient expectation of receiving a prescription, can lead to inappropriate antibiotic prescriptions.^7–10^

Clinicians often rely on medical history and physical exams to determine if antibiotics are warranted, but physical examination alone has poor accuracy for diagnosis of ARIs.^11^ Some pathogen-specific diagnostic tests are available in outpatient settings (eg, influenza, streptococcal pharyngitis, SARS-CoV-2), but these are limited to specific conditions and there are not widely available point-of-care tests that can quickly distinguish between bacterial and viral infections.^1,12^ A rapid test that can rule out bacterial infection while reliably detecting viral infection will enable clinicians to make prompt, informed decisions about treatment and support antimicrobial stewardship.^4,13^

Simple, inexpensive tests that can guide antibiotic prescription in the outpatient setting have been identified as a successful strategy to reduce inappropriate prescription of antibiotics.^4,14,15^ FebriDx^®^ (Lumos Diagnostics, Sarasota, FL) is an example of a rapid diagnostic test that provides both the clinician and patient with information about the cause of a patient’s infection to guide appropriate decisions about antibiotic prescriptions. The primary aim of this study is to conduct a US economic evaluation of FebriDx, a novel, rapid point-of-care test that simultaneously measures two key infection biomarkers, C-reactive protein and Myxovirus resistance protein A (MxA) to accurately differentiate viral from bacterial infection.^16,17^

## INTERVENTION

FebriDx is a 10-minute, point-of-care test that uses fingerstick blood to identify a host response to a bacterial or viral infection to identify which patients may require antibiotics. C-reactive protein is a non-specific acute phase protein that is elevated in both viral and bacterial infections and MxA is elevated in acute viral infections. MxA specifically recognizes newly synthesized viral nucleoproteins or nucleocapsid proteins and forms large intracellular MxA-nucleoprotein immune complexes that accumulate and aggregate in the cytoplasm of infected cells to inhibit viral replication.^18^ When paired together, the dual biomarker technology of FebriDx improves the sensitivity and specificity of both markers to accurately and reliably differentiate viral and bacterial ARIs.^16,17,19^

The diagnostic accuracy of FebriDx to distinguish between bacterial and viral ARI has been evaluated in multi-center US trials. The FebriDx test was determined to have both high sensitivity (up to 95%) to detect a bacterial infection and up to 99% negative predictive value to safely rule out a bacterial infection.^17^ A recent economic evaluation conducted from a UK perspective found that implementation of FebriDx reduced the costs related to acute respiratory infection by approximately 27%.^20^

## METHODS

This economic evaluation was developed based on a literature review of US-based ARI studies indexed on PubMed. The search for key model inputs was restricted to English-language studies of large, real-world US datasets, published between 2010 and 2020. Costs were estimated from a payer perspective, and all costs were inflated to 2020 US dollars using the Consumer Price Index.^21^

Three key populations were considered in the economic model: (1) the US patient population diagnosed with each ARI annually; (2) the patient population with each ARI condition treated with antibiotics under standard of care; and (3) the patient population for whom antibiotic treatment was appropriate. The overall and condition-specific population sizes were estimated from large US-based outpatient studies. The model included the costs attributable to antibiotics, follow-up care, antibiotic resistance, and diagnostic testing. Patients treated with prescription antibiotics incur the direct cost of the antibiotic. A subset of patients treated with antibiotics also incur the follow-up costs associated with treating drug-related adverse events in outpatient clinics, EDs, and hospitals. Patients treated with antibiotics also incur the cost of antibiotic resistant infections, regardless of whether the antibiotic prescription was appropriate.

Patients suffering from ARIs who received antibiotics were typically prescribed penicillins, macrolides, cephalosporins, sulfonamides, or fluoroquinolones as monotherapy or combination therapy. The class of antibiotic depended on ARI condition, and each antibiotic was associated with specific adverse event rates and costs. The study assumed that all reported adverse events required follow-up treatment.

The impact of FebriDx testing on antibiotic prescription rates was estimated based on test sensitivity and specificity. FebriDx can rule out viral infection with up to 95% sensitivity and 94% specificity.^17^ The model, therefore, assumes that FebriDx identifies 95% of patients with bacterial infections who are appropriate candidates for antibiotics. Ninety-four percent of patients with viral infections are correctly identified as inappropriate candidates for antibiotics, while 6% of patients with viral infections are identified as possible bacterial infections and receive antibiotics in error.

### Patient Population

US outpatient ambulatory care centers and EDs see about 57.9 million patient visits for ARIs annually.^2^ Urgent care and retail clinics account for an additional 96 million ARI-related visits.^22^ Incidence rates, antibiotic prescription rates, and appropriateness of antibiotic prescriptions vary across type of infection and care settings. Therefore, incidence and prescription rates were estimated from three large US ARI studies to capture variation in prescribing patterns across different care settings.^23–25^ Overall incidence and prescription rates were weighted by the proportion of patients treated in each setting according to Palms et al.^23^ (**Tables S1 & S2 in the Online Supplementary Material**). The size of each patient population is presented in Table 1. These estimates of patient populations and prescribing practices provide a baseline picture of outpatient ARI care in the United States against which FebriDx-guided antibiotic prescription was compared.

**Table 1.** US Acute Respiratory Infection Prevalence and Antibiotic Prescription Rates

**Diagnosis**	**ARI Consultations by Condition^a^**		**Prescribed Antibiotics^b^**		**Appropriate Antibiotics^c^**	
	%	N	%	N	%	N
**Sinusitis**	29%	43 911 707	76%	33 534 895	2%	878 234
**Otitis Media**	11%	17 667 202	79%	14 003 932	52%	9 186 945
**Pharyngitis**	25%	38 281 229	52%	19 940 967	15%	5 742 184
**Viral Upper Respiratory Tract Infection**	22%	34 264 119	31%	10 504 956	0%	-
**Bronchitis**	8%	12 786 866	73%	9 307 467	0%	-
**Pneumonia**	2%	2 911 968	75%	2 175 468	76%	2 213 096
**Influenza**	3%	4 071 373	11%	465 173	0%	-
**Total**	100%	153 894 464	58%	89 932 858	12%	18 020 459

Abbreviations: ARI, acute respiratory infections.

^a^See Supplementary Table S1 for detail.<br>^b^See Supplementary Table S2 for detail.<br>^c^Sinusitis - Harris (2016); Otitis Media - McGrath (2013); Pharyngitis – Shapiro (2020); Viral URI, Bronchitis, Influenza – Fleming-Dutra (2016); Pneumonia – Jain (2015).

Antibiotics are necessary to treat some persistent or severe bacterial respiratory infections, but they are ineffective and inappropriate for treatment of viral infections.^25,26^ Quantifying the amount of antibiotics that are medically necessary provides an “appropriate” prescription rate, which diagnostic testing can help to achieve. Acute sinusitis infections are commonly caused by viruses, allergies, or other irritants, and antibiotics are necessary in less than 2% of cases.^1^ Acute otitis media may be caused by either viruses or bacteria. A 2013 study of otitis media found that antibiotic prescription rates dropped to 52% after implementation of antibiotic prescribing guidelines, suggesting that prescription rates higher than 52% are inappropriate.^27^ Pharyngitis can also be caused by either viruses or bacteria, and an antibiotic prescription is only indicated for Group A Streptococcal infections, which occur in 5-15% of adult pharyngitis cases.^1,28^ Community-acquired pneumonia can be caused by bacteria or viruses. A 2015 study evaluating the epidemiology of community-acquired pneumonia found that 24% of pneumonia cases were conclusively attributable to viral pathogens. The remaining 76% of infections were attributable to bacterial infection (11%), bacterial-viral coinfection (3%), fungal or mycobacterial infection (1%), or an unidentified pathogen (62%).^29^ Therefore, antibiotics could be appropriate in up to 76% of pneumonia cases. The estimated number of patients with each ARI that would benefit from appropriate antibiotic treatment are estimated in the final two columns of Table 1.

### Antibiotic Type

ARIs are commonly treated with penicillin, macrolide, and cephalosporin antibiotics. Fluroquinolone, sulfonamide, and tetracycline antibiotics are generally reserved for patients with a penicillin allergy.^1^ Each antibiotic has a different cost and adverse event rate. To account for these differences, the class of antibiotics prescribed for each ARI (including antibiotics inappropriately prescribed for viral ARIs) were estimated from two studies.^3,26^ This distribution was incorporated into calculations in order to account for differences in adverse event rates and the direct costs associated with each antibiotic (Table 2).^30–33^

**Table 2.** Overall Probability of Adverse Events by Antibiotic Type and Care Setting

	**Outpatient**	**Emergency Department**
Penicillins	1.30%	0.62%
Macrolides	0.19%	0.43%
Cephalosporins	0.52%	0.32%
Quinolones	0.53%	0.47%
Sulfonamides/Tetracyclines	1.80%	0.84%
Other	3.69%	0.30%
**Total (11%)**	**8.02%**	**2.98%**

Notes: Estimated from Bourgeois et al. (2010)^30^ and Geller et al. (2018).^31^

### Adverse Events

Patients treated with antibiotics experience a variety of adverse events including gastrointestinal distress, rash, anaphylaxis, and Stevens-Johnson syndrome.^30,34^ A 2020 study of antibiotic-related adverse reactions found that 11% of patients treated with antibiotics in an outpatient setting experienced adverse reactions.^34^ The study was a retrospective chart review, therefore, the model assumes that all adverse reactions reported in the study were significant enough to require follow-up care, though this may be an overestimation that is accounted for in the sensitivity analysis. Patients experiencing more mild adverse events are more likely to seek follow-up care in an outpatient setting, while emergent reactions like anaphylaxis and Stevens-Johnson syndrome are treated in the ED.

A longitudinal study of adverse drug events found that 73% of patients experiencing antibiotic-associated adverse events sought care in an outpatient clinic, while the remainder were treated in the ED.^30^ Accounting for the distribution of total adverse events (11%) across settings for follow-up care, we estimate that 8% of all patients treated with antibiotics required follow-up care in an outpatient clinic, and 3% received care in the ED.^34^ A 2018 study by Geller et al. estimated that 9% of adverse event-related ED visits require hospitalization.^31^ The overall probability of an ED visit requiring hospitalization was, therefore, 0.07%.

Each antibiotic had a unique distribution of adverse events, which occurred at different rates. For example, penicillin and sulfonamide antibiotics accounted for a larger proportion of adverse reactions than other antibiotic types.^30,34^ Antibiotic-specific adverse event rates were estimated from two national studies of adverse drug events in EDs and outpatient clinics (Table 2).^30,31^

### Costs

There are several types of antibiotics included in each class, but azithromycin, amoxicillin, amoxicillin-clavulanate, doxycycline, and levofloxacin were among the most common antibiotics prescribed for ARIs.^1,26^ According to guidelines approved by the Centers for Disease Control and the American College of Physicians, appropriate treatment lengths for bacterial ARIs range from 5-10 days.^1^ Total antibiotic costs depended on the total number of tablets or capsules required for a full treatment cycle. For example, guidelines recommend that acute rhinosinusitis is treated with 500 mg amoxicillin and 125 mg clavulanate 3 times daily for 5-7 days, which requires 15-21 tablets.^1^ The longest treatment regimen was chosen for model estimation. The cost of each antibiotic was estimated from the 2019 Medical Expenditure Panel Survey Prescribed Medicines file by calculating the average payer cost for each antibiotic at the appropriate dose and total number of tablets or capsules (Table 3).^35^

**Table 3.** Office, Emergency Room, Hospital, Antibiotic Costs^a^

**Costs**	**Value**	**Dose**
FebriDx^b^	$23	
Office-based Outpatient Visit	$245	
Emergency Department Visit^c,^^36^	$1156	
Hospitalization^d,^^37^	$14 678	
Antibiotic Resistance^e,^^38^	$49	
Penicillin Antibiotic^35^	$10.65	Penicillin 500mg 2x daily for 10 days, amoxicillin 500mg 3x daily for 10 days, amoxicillin 500mg/clavulanate 125 mg 3x daily for 7 days.^1^^,f^
Macrolide Antibiotic^35^	$19.55	Azithromycin 250mg 1x daily for 7 days.^1^
Cephalosporin Antibiotic^35^	$36.27	Cephalexin 500mg 2x daily for 10 days.^1^
Sulfonamide/tetracycline Antibiotic^35^	$24.73	Doxycycline 100mg 2x daily for 7 days.^1^
Fluoroquinolone Antibiotic^35^	$42.65	Levofloxacin 500mg 1x daily for 7 days.^1^

^a^Costs expressed in 2020 USD.<br>^b^Estimate.<br>^c^Does not include cost of hospital admission.<br>^d^Adverse drug event present on admission.<br>^e^Estimated cost of antibiotic resistance per outpatient antibiotic prescribed.<br>^f^79% amoxicillin, 17% amoxicillin-clavulanate, 4% penicillin.^26^

The cost of second-line inappropriate antibiotics for viral conditions were also included when estimating the total cost of antibiotic treatment. Antibiotics are ineffective for viral conditions, and 8.9% of viral ARI patients return for a second antibiotic prescription when their symptoms are not resolved by the first antibiotic.^3^ Patients treated with antibiotics often experience a drug related adverse event, some of which require follow-up care.^34^ Follow-up outpatient visits costs approximately US 245/visit,andafollow−upEDvisitcostsapproximatelyUS1156/visit^36^ (Table 3). Hospitalization due to an adverse drug event present on admission was estimated to cost US 14 678.[@87672]Inaddition,itwasassumedthatallpatientsexposedtoantibiotictreatmenthadanincreasedriskofdevelopinganantibioticresistantinfection.ThiscostwasdeterminedbasedonastudybyMichaelidisetal.thatfoundthattheestimatedcostofantibioticresistancewasUS49 per outpatient antibiotic prescribed for an ARI.^38^ In the United States, FebriDx was assumed to be reimbursed under the Centers for Medicare and Medicaid Services (CMS) Clinical Laboratory Fee Schedule. CMS has prioritized supporting antimicrobial stewardship efforts. For example, CMS expanded the intended use of Procalcitonin (Code 8414, CMS reimbursement of US 27),astandalonebiomarkerusedtomanagepatientswithsuspectedbacterialinfection,tosupportitsuseasanaidtoreduceunnecessaryantibioticprescriptions.[@87674]Atthetimeofpublication,FebriDxhasnotbeenassignedareimbursementcode,butbasedonotherbiomarkers/chemistrytestswithestablishedreimbursementcodes,itisreasonabletoassumeaconservativereimbursementofapproximatelyUS18.40-27.60wouldbeassigned.Theaverageoftherange(US23) was used as a base case for reimbursement (Table 3). Based on reimbursement ranging from US 18.40−27.60, end users’ costs will be covered to account for their time and administrative costs to perform the test.

The total expected cost of antibiotic treatment for ARIs was a weighted sum of the product of each cost and the probability of incurring the cost, calculated as follows:

$$\begin{array}{rl} E(\$)= & P(outpt.AE)\times \$(outpt.visit)\\ & +P(ERAE)\times \$(ERvisit)\\ & +P(hosp.)\times \$(hosp.) \end{array}$$

where (outpatientvisit)=US245, $(ED visit)=US 1156,and(hospitalization)=US $14 679. The probability of each event was specific to the condition and antibiotic type as described in Table 2.

## RESULTS

**Figure 1.**
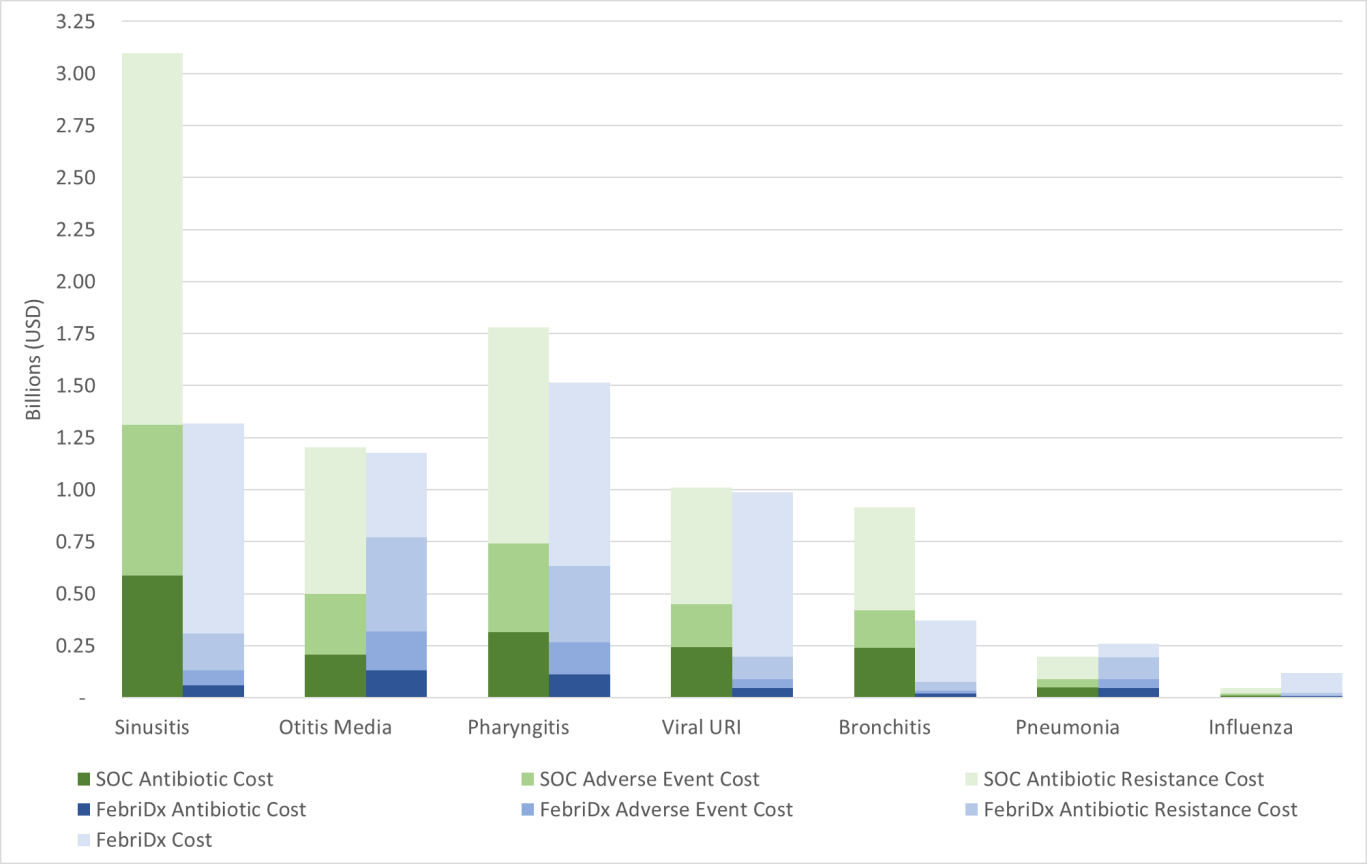
Estimated Costs by Acute Respiratory Infection (ARI) Type

The expected national cost to treat ARIs under standard of care was US 8.25billion,includingthecostofantibiotics,antibiotic−relatedadverseevents,andantibioticresistance.UsingapresumedMedicarereimbursementofUS23, the expected national cost associated with a FebriDx guided ARI treatment was US 5.74billionandthesavingsassociatedwiththeuseofFebriDxtotaledUS2.51 billion. The benefit of FebriDx varied depending on the type of ARI (Figure 1).

**Figure 2.**
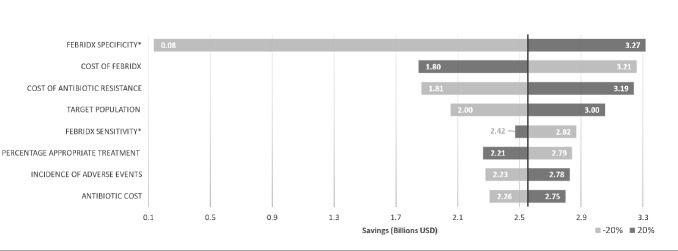
Sensitivity Analysis *Maximum=1.

A sensitivity analysis was conducted to assess the cost drivers of the model by varying each parameter by 20% (Figure 2) and resulted in a total expected cost savings that ranged from US 0.08toUS3.27 billion. The national cost is strongly influenced by the sensitivity of the FebriDx test because its results determine the economic impact of further treatment. The sensitivity analysis tested two scenarios; one in which FebriDx was assumed to have a sensitivity of 75%, and the other in which FebriDx was assumed to have a sensitivity of 100%. In clinical studies, the diagnostic performance of FebriDx has ranged from 85-95% sensitivity and 83-94% specificity.^17,39^

The model is also sensitive to the reimbursed cost of FebriDx and the size of the population seeking treatment for ARIs. The cost of antibiotic resistance also has an impact because 100% of patients exposed to antibiotics incur the risk and the expected cost of an antibiotic resistant infection. Incidence of adverse events has a less significant impact on the model because although adverse events add considerably to cost of care, relatively few (11.02%) patients experienced adverse events that required follow-up care.^34^

## DISCUSSION

Antibiotic stewardship efforts have increased in the United States in recent years, driven by the human and economic cost of antibiotic-resistant infections and adverse events.^4^ Outpatient settings are an important focus for antibiotic stewardship as they accounted for 59% of all antibiotic expenditures in the United States from 2010 to 2015.^40^ Outpatient antibiotic prescription rates for ARIs are highest in urgent care centers and EDs,^23^ and are often driven by fear of missing a bacterial infection, clinician perception that a patient expects an antibiotic, and/or direct patient requests.^41^ FebriDx, a rapid diagnostic test, addresses this issue by arming clinicians with actionable information regarding the cause of infection at the point of care (ie, during the patient visit) to guide antibiotic prescription and communication with the patient about the necessity of antibiotics.

Although these results are encouraging, there are limitations to this model that should be considered. This model is literature-based and, therefore, there is some inherent uncertainty in model parameters. Although this uncertainty was mitigated by estimating parameters from large national studies where possible, the parameters are not all nationally representative. The real-world performance of diagnostics can also be influenced by patient characteristics, including the length of symptoms and the severity of the ARI, as well as disease prevalence. The model does not consider costs of “missed” bacterial infections because delaying antibiotics or using a “watchful waiting” approach does not significantly increase complication rates in most acute respiratory infections managed in an outpatient setting.^42–45^ Pneumonia, which accounts for approximately 2% of outpatient ARI visits,^23–25^ is an exception in some cases because severe infection can be life-threatening, particularly for older or chronically ill patients.^46^ The model also does not consider the costs associated with antiviral medications because most cases of viral respiratory infection are self-limiting and do not require antiviral medications.^1,47^ Antivirals are indicated to treat influenza if patients have severe symptoms, present to a health-care provider within the window of therapeutic benefit, are older than 65, pregnant, or have other risk factors.^48^ Supportive therapies for symptomatic relief including over-the-counter medications such as decongestants, antihistamines, and cough suppressants, are often used for both bacterial and viral respiratory infections.^1^

This model demonstrates that use of FebriDx in the outpatient setting can lead to a considerable reduction in unnecessary antibiotic prescriptions, which will in turn lead to a reduction in antimicrobial resistance and adverse events. The model does not consider the benefits of enhanced workflow and efficiency in emergency and urgent care settings, rapid identification of bacterial infection, and the reduced risk of increased morbidity or sepsis associated with delayed treatment, nor does this model include costs of other testing and acceleration of antimicrobial resistance associated with the ongoing COVID-19 pandemic. Preventing antimicrobial resistance not only saves health-care costs, it also can prevent loss of life. It is estimated that 10 million people will die due to drug-resistant infection by 2050 if action is not taken to reduce misuse of antimicrobials.^49^ The cost savings in this analysis are driven in part by avoided costs of antibiotic resistance. Rapid diagnostics that aid in the appropriate use of antibiotics will curb the rising rates of resistant infections, which can prevent the development of further resistance and, therefore, can avert fatalities and reduce future health-care costs.^4^

## CONCLUSION

FebriDx is a simple, rapid point-of-care test that aids in the differentiation of viral from bacterial ARIs reducing the misuse of antibiotics and, therefore, combatting the growing threat of antibiotic resistance. FebriDx-guided ARI diagnosis demonstrated a US $2.51 billion cost savings compared to the standard of care approach of guiding antibiotic treatment of ARIs in US outpatient settings. The cost savings were driven by the reduction in antibiotic resistance associated with a lower antibiotic prescription rate as well as avoided return visits for drug related adverse events.

## Supplementary Material

Online Supplementary Material
