# Supplementary material for: Economic Evaluation of FebriDx®: A Novel Rapid, Point-of-Care Test for Differentiation of Viral versus Bacterial Acute Respiratory Infection in the United States
Source: J Health Econ Outcomes Res. 2021 Sep 30;8(2):56–62. doi: 10.36469/001c.27753 (PMC8483888; doi:10.36469/001c.27753)
Supplement: Online Supplementary Material [file jheor_2021_8_2_27753_72498.pdf]

Journal of Health Economics and Outcomes Research

# Online Supplementary Material

Dick K, Schneider J. Economic evaluation of FebriDx®: A novel rapid, point-of-care test for differentiation of viral versus bacterial acute respiratory infection in the United States. *JHEOR*. 2021;8(2)56-62.
doi:10.36469/jheor.2021.27753

**Table S1.** ARI Consultations by Condition Across Care Settings
**Table S2.** Prescribed Antibiotics by Condition Across Care Settings

This supplementary material has been provided by the authors to give readers additional information about their work.

This is an open-access article distributed under the terms of the Creative Commons Attribution 4.0 International License (CCBY-4.0). View this license's legal deed at http://creativecommons.org/licenses/by/4.0 and legal code at http://creativecommons.org/licenses/by/4.0/legalcode for more information.

**Table S1. ARI Consultations by Condition Across Care Settings**

| Care Setting | Urgent Care[a] | Retail Clinic[a] | Emergency Department[b] | Ambulatory Care Center[c] | Weighted Average Across Settings[d] |
|---|---|---|---|---|---|
| Sinusitis | 27% | 32% | 15% | 29% | 29% |
| Otitis Media | 9% | 11% | 16% | 12% | 11% |
| Pharyngitis | 29% | 29% | 4% | 25% | 25% |
| Viral Upper Respiratory Tract Infection | 19% | 18% | 29% | 22% | 22% |
| Bronchitis | 11% | 5% | 18% | 8% | 8% |
| Pneumonia | 2% | 0% | 10% | 2% | 2% |
| Influenza | 4% | 4% | 8% | 2% | 3% |

[a]Palms (2018).
[b]Weighted average Palms (2018) and Donnelly (2014).
[c]Weighted average Palms (2018) and Fleming-Dutra (2016).
[d]Weighted based on distribution of ARI patients by setting in Palms (2018) – 9% urgent care, 0.25% retail clinic, 3% emergency department, 88% ambulatory care center.

**Table S2. Prescribed Antibiotics by Condition Across Care Settings**

| | Urgent Care[a] | Retail Clinic[a] | Emergency Department[b] | Ambulatory Care Center[c] | Weighted Average Across Settings[d] |
|---|---|---|---|---|---|
| Sinusitis | 82% | 87% | 68% | 76% | 76% |
| Otitis Media | 83% | 86% | 73% | 79% | 79% |
| Pharyngitis | 60% | 57% | 50% | 51% | 52% |
| Viral Upper Respiratory Tract Infection | 42% | 11% | 20% | 30% | 31% |
| Bronchitis | 76% | 31% | 57% | 73% | 73% |
| Pneumonia | 72% | 91% | 67% | 75% | 75% |
| Influenza | 13% | 4% | 8% | 11% | 11% |
| Total | 64% | 58% | 48% | 58% | 58% |

[a]Palms (2018).
[b]Weighted average Palms (2018) and Donnelly (2014).
[c]Weighted average Palms (2018) and Fleming-Dutra (2016).
[d]Weighted based on distribution of ARI patients by setting in Palms (2018) - 9% urgent care, 0.25% retail clinic, 3% emergency department, 88% ambulatory care center.
